# Low-Intensity Physical Exercise is Associated with Improved Myelination and Reduced Microglial Activation in a Cuprizone-Induced Demyelination Model

**DOI:** 10.1007/s11064-025-04441-8

**Published:** 2025-06-05

**Authors:** Kyu Ri Hahn, In Koo Hwang, Dae Young Yoo

**Affiliations:** Department of Anatomy and Cell Biology, College of Veterinary Medicine, Research Institute for Veterinary Science, Seoul National University, Seoul, 08826 South Korea

**Keywords:** Neurogenesis, Cuprizone, Low-intensity exercise, Remyelination

## Abstract

Demyelinating diseases like multiple sclerosis cause damage to the myelin sheath, leading to neurological problems. While the exact causes of MS are unclear, it is known that inflammatory processes and poor remyelination contribute to disease progression. Exercise has shown promise as a non-drug treatment for MS, with benefits reported for mobility, mood, and potential neuroprotection. However, the specific ways in which exercise affects remyelination and neuroinflammation in demyelinating conditions are not fully understood. This study explores the effects of low-intensity physical exercise on myelination, neuroinflammation, and neurogenesis in a cuprizone-induced demyelination model, focusing on the hippocampus, which are critical for cognitive function and interhemispheric communication. Mice subjected to cuprizone treatment underwent a low-intensity forced wheel-running exercise. The results showed that low-intensity physical exercise significantly increased the expression of myelin basic protein in the stratum lacunosum-moleculare of the hippocampus and the corpus callosum, suggesting enhanced remyelination in these regions. Additionally, cuprizone-induced demyelination led to morphological changes in microglia, activating them in the hippocampus. However, low-intensity physical exercise significantly reduced microglial activation, indicating that exercise modulated the neuroinflammatory response. Despite observing reduced microglial activation with low-intensity exercise, TNF-α levels remained elevated in the low-intensity exercise group, suggesting a complex relationship between microglial activation markers and cytokine production in this model of demyelination. This indicates that low-intensity exercise may not fully suppress the pro-inflammatory potential of microglia in the cuprizone model. Although low-intensity exercise promoted remyelination and modulated neuroinflammation in the cuprizone-induced demyelination model, it did not significantly counteract the cuprizone-induced reduction in proliferating cells and immature neurons in the subgranular zone of the dentate gyrus. These findings suggest that while the exercise regimen had beneficial effects, it did not significantly influence overall neurogenesis. This novel study investigates the region-specific effects of low-intensity exercise on myelination and neuroinflammation, with a focus on the hippocampus, which is less frequently explored in the context of demyelination models. The findings highlight the potential rehabilitative benefits of low-intensity exercise for demyelination-related neurological disorders and provide new insights into the underlying mechanisms contributing to neuroprotection.

## Introduction

Demyelination and neuroinflammation are key pathological features of various neurodegenerative diseases, leading to impaired neuronal function and subsequent neurological deficits. Understanding the mechanisms underlying these processes is crucial for developing effective therapeutic strategies. The cuprizone model serves as a valuable tool for studying demyelination and neuroinflammation due to its ability to induce reproducible myelin loss in specific brain regions without triggering an autoimmune response [1]. This model, which simulates demyelination in brain white matter diseases such as multiple sclerosis (MS), involves administering cuprizone for approximately 4 to 6 weeks. During this period, characteristic pathological changes, including oligodendrocyte death, microglial activation, and astrogliosis, occur in the corpus callosum, mimicking aspects of MS lesions. Additionally, following the cessation of cuprizone administration, this model is widely used to study the remyelination process [2]. MS is closely associated with morphological and functional changes in the hippocampus, a brain region crucial for memory formation. Notably, patients with MS often exhibit significant hippocampal atrophy, along with distinct morphological alterations [3]. Additionally, substantial neuronal loss in specific hippocampal regions (CA1-3) contributes to memory impairments [4].

Exercise has emerged as a promising non-pharmacological approach for modulating neuroinflammation and promoting neuroprotection in neurodegenerative diseases. Recent studies suggest that low-intensity exercise can reduce pro-inflammatory cytokines and enhance anti-inflammatory mediators, contributing to a shift toward an anti-inflammatory state [5–7]. Mechanistically, such exercise regulates microglial activation and inhibits NF-κB pathway-mediated neuroinflammation, processes that are critically involved in demyelinating diseases like.

MS [8–10]. These findings provide a strong rationale for investigating low-intensity exercise as a potential intervention in MS models.

In addition to its anti-inflammatory effects, exercise also improves blood circulation [11], enhances metabolic activity [12], supports neural development [13]. and stimulates adult hippocampal neurogenesis [14, 15]. It elevates neurotrophic factors such as BDNF, promotes angiogenesis, and reduces oxidative stress [16]. These multimodal benefits support the growing use of exercise as an adjunct therapy for MS, with demonstrated effects on physical function, cognition, and overall quality of life [17]. Notably, exercise has also been shown to mitigate hippocampal atrophy and improve memory performance in MS patients [18, 19]. This study investigates the effects of low-intensity wheel running on myelination and neuroinflammatory processes in the hippocampus of cuprizone-treated mice. We hypothesize that even low-intensity physical exercise could positively influence myelin formation and neurogenesis in the murine hippocampus. Following the exercise period, the animals were anesthetized, sacrificed, and their brain tissues were analyzed using immunohistochemistry. Our findings contribute to the growing body of evidence supporting the neuroprotective benefits of exercise in demyelinating conditions and highlight the potential therapeutic role of low-intensity exercise in managing neurodegenerative diseases.

## Materials and Methods

### Animals

In this study, C57BL/6 N male mice were obtained from Central Lab Animal Inc. (Seoul, South Korea). The five-week-old mice were housed under controlled conditions at a constant temperature of 22 ± 2 °C with a 12-hour light-dark cycle. They were housed in groups of five per enclosure and had unrestricted access to food and water throughout the experiment. All experimental procedures were approved by the Institutional Animal Care and Use Committee of Seoul National University (SNU-171201-3) in accordance with established ethical guidelines.

**Fig. 1:**
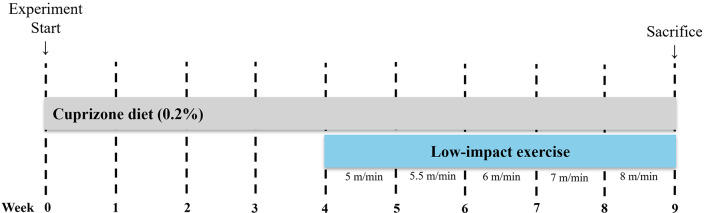
Experimental timeline. C57BL/6 mice were fed a cuprizone diet for nine weeks to induce demyelination, followed by a five-week exercise intervention with access to low-intensity running wheels

The animals were randomly divided into three groups: a control group (CTL; *n* = 10), a group fed chow containing 0.2% (w/w) cuprizone (CPZ; *n* = 10), and a group that exercised on a running wheel while consuming chow containing 0.2% cuprizone (CPZ + Ex; *n* = 10). The CPZ + Ex group received a cuprizone-containing diet for four weeks, followed by five weeks of running wheel exercise, during which the cuprizone-containing diet was maintained. Diets formulated with CPZ were prepared by incorporating 0.2% cuprizone (Sigma-Aldrich, St. Louis, MO, USA) into the AIN-76 basal feed, as described in a previous study [20]. The experimental design is summarized in Fig. 1, which offers a visual overview of the animal schedule, detailing the duration of cuprizone exposure and the exercise intervention.

### Exercise Protocol

Mice in the exercise group underwent a low-intensity forced wheel-running protocol for five weeks. To minimize physiological stress and potential confounding stress-related factors, exercise intensity was defined as speeds below 10 m/min. This definition was based on prior studies demonstrating that this range minimizes stress while maintaining physiological engagement [21, 22].

Prior to the initiation of the exercise protocol, mice were housed individually to allow accurate monitoring and control of their physical activity levels throughout the study. Mice had access to a running wheel for 30 min daily. The exercise regimen began at a speed of 5 m/min, and was gradually increased to 8 m/min by the final week to facilitate adaptation and maintain consistent exercise participation. The exercise protocol of 30 min/day for five weeks was selected based on preclinical evidence showing that this duration and frequency effectively induce beneficial effects on systemic and neurological health without causing undue strain [23]. This approach allowed for an assessment of the effects of low-intensity forced wheel running on cuprizone-induced demyelination while reducing potential confounding effects of stress.

### Tissue Preparation and Immunohistochemical/Immunofluorescent Staining

After the final running wheel session, mice were deeply anesthetized with a combination of alfaxalone (75 mg/kg; Careside, Seongnam, South Korea) and xylazine (10 mg/kg; Bayer Korea, Seoul, South Korea). Transcardial perfusion was then performed using 0.1 M phosphate-buffered saline (PBS, pH 7.4), followed by fixation with 4% paraformaldehyde (PFA) to preserve the brain tissues. Extracted brains were post-fixed in the same fixative for 12 h at 4 °C and subsequently cryoprotected by immersion in 30% sucrose in PBS for 24 h. Coronal Sect. (30 μm thickness) were obtained using a cryostat (Leica Microsystems, Wetzlar, Germany) and stored in 0.01 M PBS at 4 °C until further processing.

For immunohistochemical staining, free-floating sections were incubated with the following primary antibodies: rabbit anti-Ki67 (1:1,000; Abcam, UK, #ab15580), mouse anti-doublecortin (DCX, 1:200; Santa Cruz Biotechnology, USA, #sc-271390), rabbit anti-myelin basic protein (MBP, 1:1,000; Abcam, #ab40390), rabbit anti-ionized calcium-binding adapter molecule 1 (Iba1, 1:500; Wako, Japan, #019-19741), and mouse anti-NeuN (1:1,000; Millipore, USA). For immunofluorescent double labeling, sections were incubated with mouse anti-Iba1 (1:500; Synaptic Systems, Germany, #234011) and rabbit anti-TNF-α (1:300; Cell Signaling Technology, USA, #11948). Fluorescence images were acquired using a Leica DMi8 Thunder confocal microscope (Leica Microsystems, Germany). All procedures were carried out under consistent conditions to ensure comparability across groups.

### Quantitative Analysis and Statistical Analysis

Immunoreactive structures were quantified using ImageJ software (NIH, USA). For each animal, four coronal brain sections were analyzed, spaced 180 μm apart and spanning the region between 1.70 and 2.46 mm caudal to bregma. Images were acquired using a BX51 light microscope (Olympus, Japan) equipped with a DP71 digital camera. The immunoreactivity of DCX, MBP, TNF-α, and Iba-1 was quantified by calculating the product of pixel number and pixel density within the region of interest. Ki67-positive nuclei within the subgranular zone of the dentate gyrus (as defined by the granule cell layer) were manually counted.

All data are presented as mean ± standard deviation (SD). Immunoreactivity values for DCX, MBP, and Iba-1 were normalized to the corresponding control group and expressed as relative optical densities (RODs). Ki67-positive cell counts are reported as absolute numbers.

Statistical analyses were performed using GraphPad Prism 9.0 (GraphPad Software, USA). The Shapiro–Wilk test was used to assess data normality. For datasets meeting the assumption of normality (*p* > 0.05), parametric tests (one-way ANOVA followed by Tukey’s post-hoc test) were applied. For non-normally distributed data, non-parametric alternatives were employed. All immunohistochemical markers were analyzed using one-way ANOVA followed by Tukey’s post-hoc test for multiple comparisons. Body weight changes over time were evaluated using two-way repeated-measures ANOVA, followed by Tukey’s post-hoc test. Percentage changes in body weight were analyzed using one-way ANOVA with post-hoc comparisons. A p-value of < 0.05 was considered statistically significant.

### Colocalization Analysis

Brain tissue sections were double-stained with antibodies against TNF-alpha and Iba1, and confocal z-stack images were acquired using a Leica DMi8 Thunder microscope (Leica Microsystems, Germany). Image processing and analysis were performed using Fiji (ImageJ, NIH, USA). Regions of interest (ROIs), including the hippocampus, were manually defined, and colocalization analysis was conducted using the Coloc2 plugin.

Automatic thresholding was applied based on Costes’ method, and Manders’ tM1 coefficient, indicating the proportion of TFN-α signal colocalized with Iba1, was used as the primary metric. tM1 values were converted to percentages by multiplying by 100. For each experimental group (CTL, CPZ, CPZ + Ex), colocalization percentages were obtained from five independent biological replicates and averaged.

Statistical analysis was performed using one-way ANOVA followed by Tukey’s post-hoc test in GraphPad Prism 9.0 (GraphPad Software, USA). Data are presented as mean ± SD, and a p-value of < 0.05 was considered statistically significant.

## Result

### Changes in Body Weight Following Low-intensity Physical Exercise in Cuprizone-Treated Mice

**Fig. 2:**
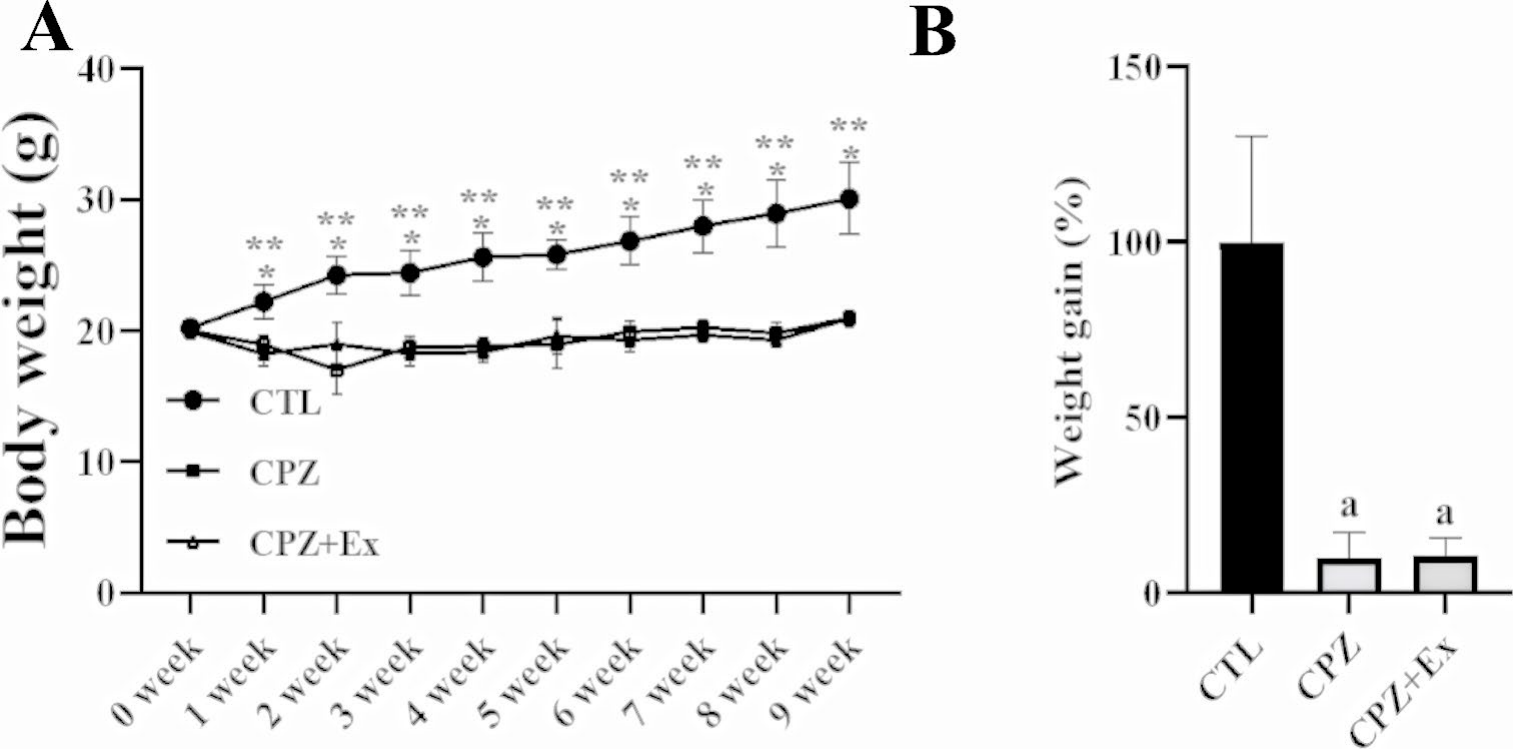
Body weight changes in Control, CPZ (cuprizone-treated), and CPZ + Ex (cuprizone-treated with exercise) groups over time. (**A**) The graph illustrates the changes in body weight among three groups of mice throughout the experiment. (**B**) The groups exhibited varying degrees of weight gain, measured as the percentage change from their initial body weights over the 9-week period. CTL group showed a consistent increase in body weight over time. In contrast, the CPZ group and CPZ + Ex group did not display significant weight gain after cuprizone administration. Tukey’s multiple comparisons test revealed that the control group had a significantly higher mean body weight than both the CPZ group and the CPZ + Ex group. However, no significant difference was observed between the CPZ and CPZ + Ex groups (*p* > 0.05). *: *p* < 0.05 for CTL vs. CPZ, **: *p* < 0.05 for CTL vs. CPZ + Ex. CTL vs. CPZ *p* < 0.05^a^*p* < 0.05 vs. CTL; ^b^*p* < 0.05 vs. CPZ. The error bars represent the standard deviation of the mean

The control group exhibited a steady increase in body weight over time, whereas body weights in the CPZ and CPZ + Ex groups remained relatively unchanged following cuprizone administration (Fig. 2A). The control group maintained a significantly higher average weekly weight compared to the other experimental groups (Two-way ANOVA, group effect: F = 382.9, *p* < 0.0001). These results indicate that cuprizone treatment had a substantial impact on body weight gain. Specifically, the significant weight loss observed in the CPZ groups supports the expected systemic effects of cuprizone, suggesting that exercise had a limited influence on body weight in this context. Additionally, no significant difference in body weight was observed between the CPZ and CPZ + Ex groups at any time point.

Similarly, the percentage of body weight gain relative to initial weight was significantly greater in the control group (Fig. 2B). In contrast, the CPZ and CPZ + Ex groups exhibited weight gain percentages of 9.9% and 10.53%, respectively. These values were significantly lower than that of the control group (One-way ANOVA: F = 41.08, *p* < 0.0001).

### Changes in Myelin Basic Protein Expression in the Hippocampus Following Low-Intensity Exercise in Cuprizone-Treated Mice

**Fig. 3:**
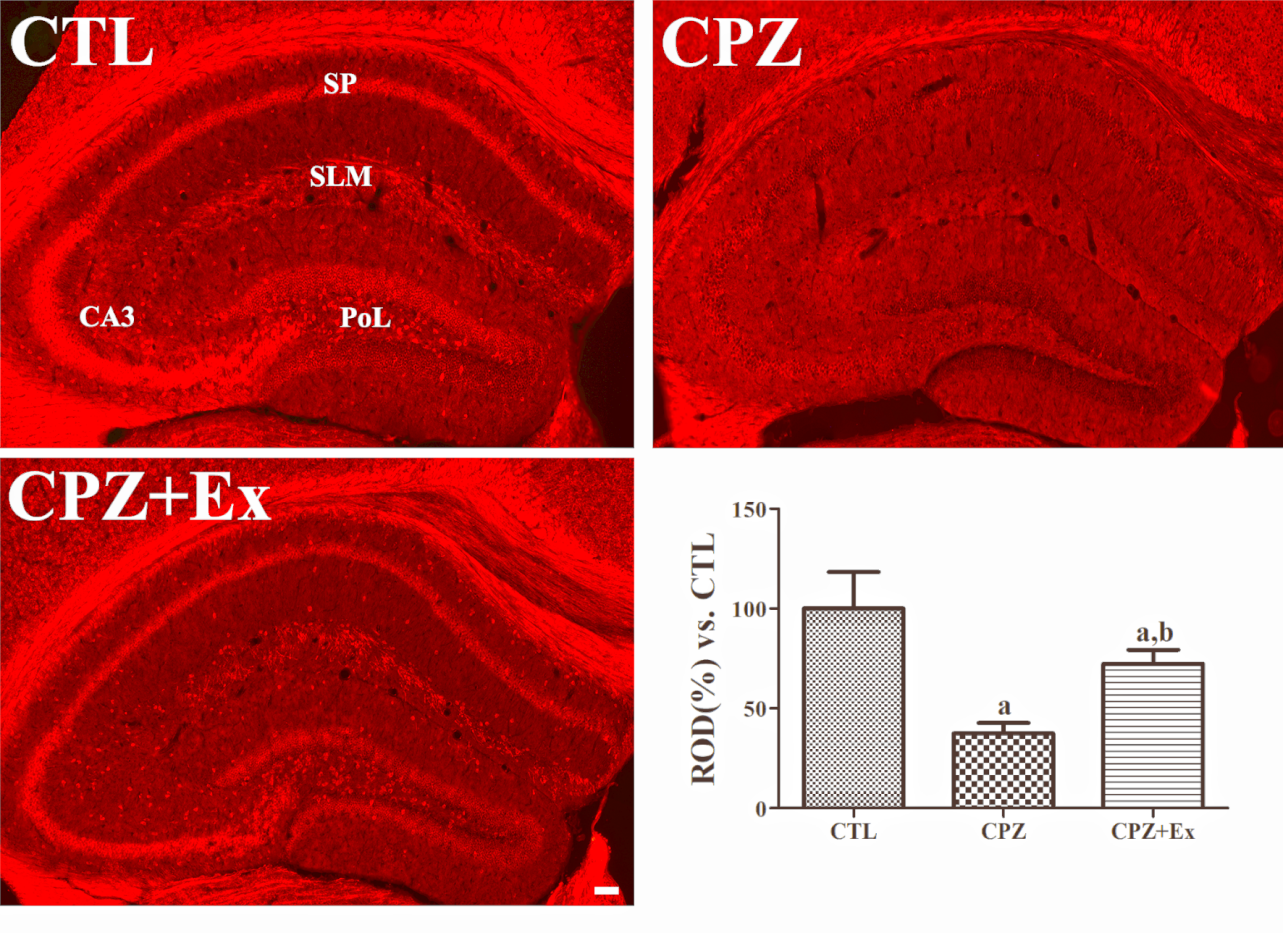
Immunohistochemical staining for myelin basic protein (MBP) in the hippocampus of control (CTL), cuprizone-fed (CPZ), and cuprizone-fed with exercise (CPZ + Ex) groups. SP: stratum pyramidale; SLM: stratum lacunosum-moleculare; CA3: Cornu Ammonis 3; CC: corpus callosum; PoL: polymorphic layer. Scale bar = 50 μm. Tukey’s multiple comparisons test revealed that the CPZ group had a significantly lower MBP immunoreactivity than CTL group. In addition, higher MBP immunoreactivity was found in the CPZ + Ex group than CPZ group. ^a^*p* < 0.05 vs. CTL; ^b^*p* < 0.05 vs. CPZ. The error bars represent the standard deviation of the mean

To assess differences in myelin protein levels, we performed immunohistochemical analysis of myelin basic protein (MBP) expression (Fig. 3). In the CTL group, MBP immunoreactivity was observed in the stratum lacunosum-moleculare (SLM), alveus, and corpus callosum, as well as in the stratum pyramidale of the CA1 and CA3 regions and the polymorphic layer of the dentate gyrus. In contrast, the CPZ group exhibited markedly reduced MBP immunoreactivity in both the hippocampus and corpus callosum, with total MBP immunoreactivity decreasing to 37.41% of the control group levels.

However, in the CPZ + Ex group, MBP immunoreactivity significantly increased compared to the CPZ group, reaching 72.35% of the control group levels. These results suggest that low-intensity physical exercise facilitates partial remyelination in cuprizone-induced demyelination.

### Changes in Microglial Morphology in the hippocampus Following low-intensity Exercise in Cuprizone-Treated Mice

**Fig. 4:**
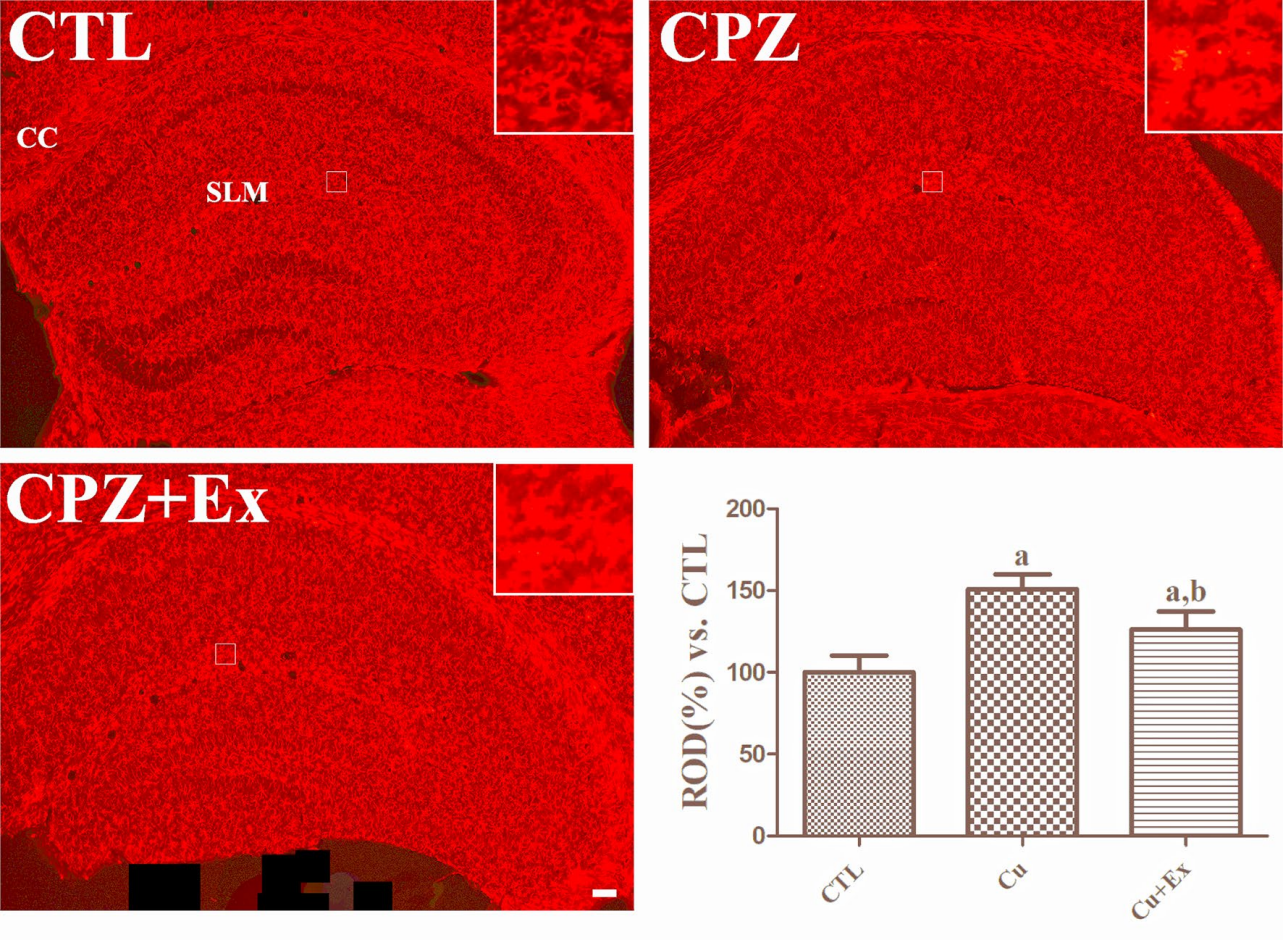
Iba1 immunofluorescence in the hippocampus. Images show Iba1-labeled cell bodies and processes (red). SLM: stratum lacunosum-moleculare; CC: corpus callosum. Scale bar = 50 μm. Tukey’s multiple comparisons test revealed that Iba1 immunoreactivity was highest in CPZ group and lowest in CTL group. ^a^*p* < 0.05 vs. CTL; ^b^*p* < 0.05 vs. CPZ. The error bars represent the standard deviation of the mean

To evaluate the effects of exercise on hippocampal microglial morphology, we conducted Iba1 immunostaining (Fig. 4). In all groups, Iba1-positive microglial cells were detected in the hippocampus and corpus callosum. In the CTL group, microglia exhibited thin processes and small cytoplasm, indicative of a resting state. In contrast, microglia in the CPZ group displayed hypertrophied cytoplasm and thickened processes in the stratum lacunosum-moleculare, polymorphic layer of the dentate gyrus, and corpus callosum, suggesting an activated state associated with neuroinflammation.

Quantitative analysis revealed that Iba1 immunoreactive levels increased by 150.90% in the CPZ group and by 126.20% in the CPZ + Ex group relative to the CTL group baseline (100%). Notably, the CPZ + Ex group showed a significant reduction in Iba1 immunoreactivity compared to the CPZ group, indicating that exercise attenuated microglial activation. 

### Changes in Microglial Morphology and TNF-α–Related Activation in the Hippocampus Following Low-intensity Exercise in Cuprizone-Treated Mice

**Fig. 5:**
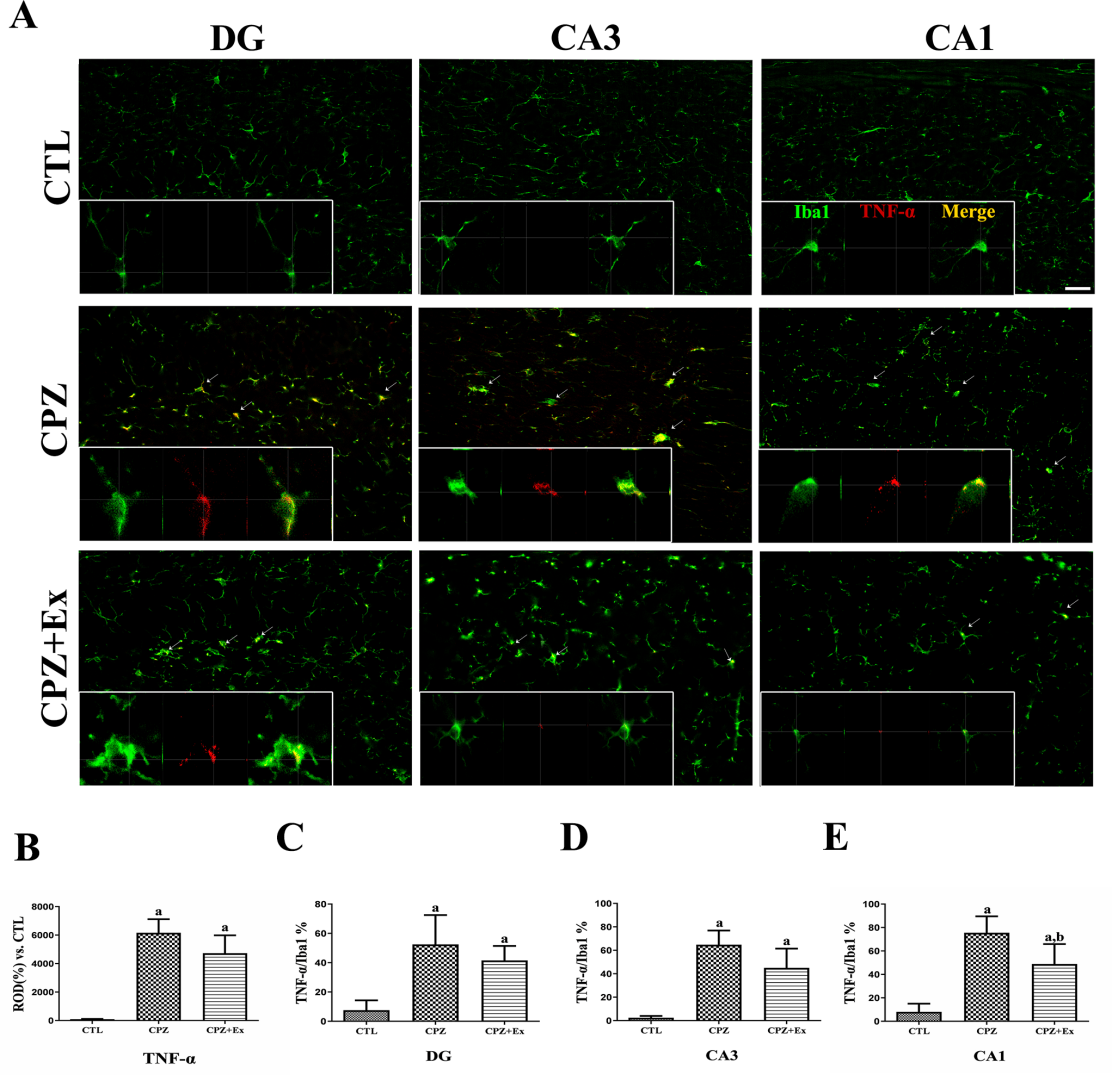
Double immunofluorescence staining and analysis of microglial activation in the hippocampus. (**A**) Representative images of double immunofluorescence staining for Iba1 (green) and TNF-α (red) in the hippocampus, taken from different subregions (DG, CA3, and CA1) across experimental groups (CTL, CPZ, CPZ + EX). Iba1⁺TNF-α⁺ double-positive microglia appear yellow in the merged images, indicating colocalization. High-magnification orthogonal views of Iba1 and TNF-α signals within individual microglia are shown below each panel to highlight spatial colocalization. Scale bar = 25 μm. (**B**) Quantitative analysis of TNF-α expression in the hippocampus across experimental groups (CTL, CPZ, CPZ + EX), illustrating regional and group-specific differences in pro-inflammatory signaling. TNF-α is a key pro-inflammatory cytokine released by activated microglia. (**C**–**E**) Quantification of colocalization between Iba1 and TNF-α signals in each hippocampal subregion: dentate gyrus, CA3 (**D**), and CA1 (**E**). These measurements reflect the degree of microglial activation and pro-inflammatory status across the experimental conditions. ^a^*p* < 0.05 vs. CTL; ^b^*p* < 0.05 vs. CPZ. The error bars represent the standard deviation of the mean

To evaluate the spatial expression of TNF-α specifically in microglia, double immunofluorescence staining for Iba1 and a key of pro-inflammatory cytokine TNF-α was performed across hippocampal subregions. This approach enabled the identification of TNF-α expressing microglia and assessment of their activation states in response to experimental conditions. In both the CPZ and CPZ + EX groups, there was a notable increase in Iba1⁺TNF-α⁺ double-positive microglia compared to the CTL group, indicating elevated pro-inflammatory microglial activity. These cells appeared yellow in the merged images, reflecting strong colocalization of Iba1 (green) and TNF-α (red). High-magnification images in the lower panels highlighted intense TNF-α expression in hypertrophic, amoeboid microglia predominantly in the CPZ group, while the CPZ + EX group displayed moderate TNF-α signals and partially restored microglial morphology. In contrast, the CTL group showed minimal TNF-α staining, suggesting a quiescent microglial state. These findings indicate that TNF-α upregulation is a hallmark of CPZ-induced microglial activation, and that exercise attenuates this inflammatory response (Fig. 5A).

Quantitative analysis further supported these observations. TNF-α expression in the hippocampus was significantly increased in the CPZ group, reaching 6160% of the CTL baseline. The CPZ + EX group also showed elevated levels (4733%), but to a lesser extent than CPZ alone. Statistical comparisons revealed a significant difference between CTL and the other two groups (*p* < 0.05), whereas the difference between CPZ and CPZ + EX did not reach statistical significance, although a downward trend was evident (Fig. 5B).

Region-specific analysis of colocalization between TNF-α and Iba1 showed the highest colocalization in the CPZ group across all hippocampal subregions (DG, CA3, CA1) (Fig. 5C–E). In the dentate gyrus, the percentage of Iba1⁺TNF-α⁺ cells were 7.58% in the CTL group, 52.52% in CPZ, and 41.58% in CPZ + EX. In CA3, values were 2.49%, 64.82%, and 45.00%, respectively. In CA1, the values were 8.02%, 75.50%, and 48.91%. All three subregions showed a marked increase in TNF-α colocalization following CPZ treatment. While both CPZ and CPZ + EX groups showed significantly higher TNF-α/Iba1 colocalization compared to CTL, only in the CA1 region was a significant reduction observed in the CPZ + EX group compared to CPZ, suggesting region-specific anti-inflammatory effects of exercise.

### Changes in Cell Proliferation in the Hippocampal Dentate Gyrus Following Low-intensity Physical Exercise in Cuprizone-Treated Mice

**Fig. 6:**
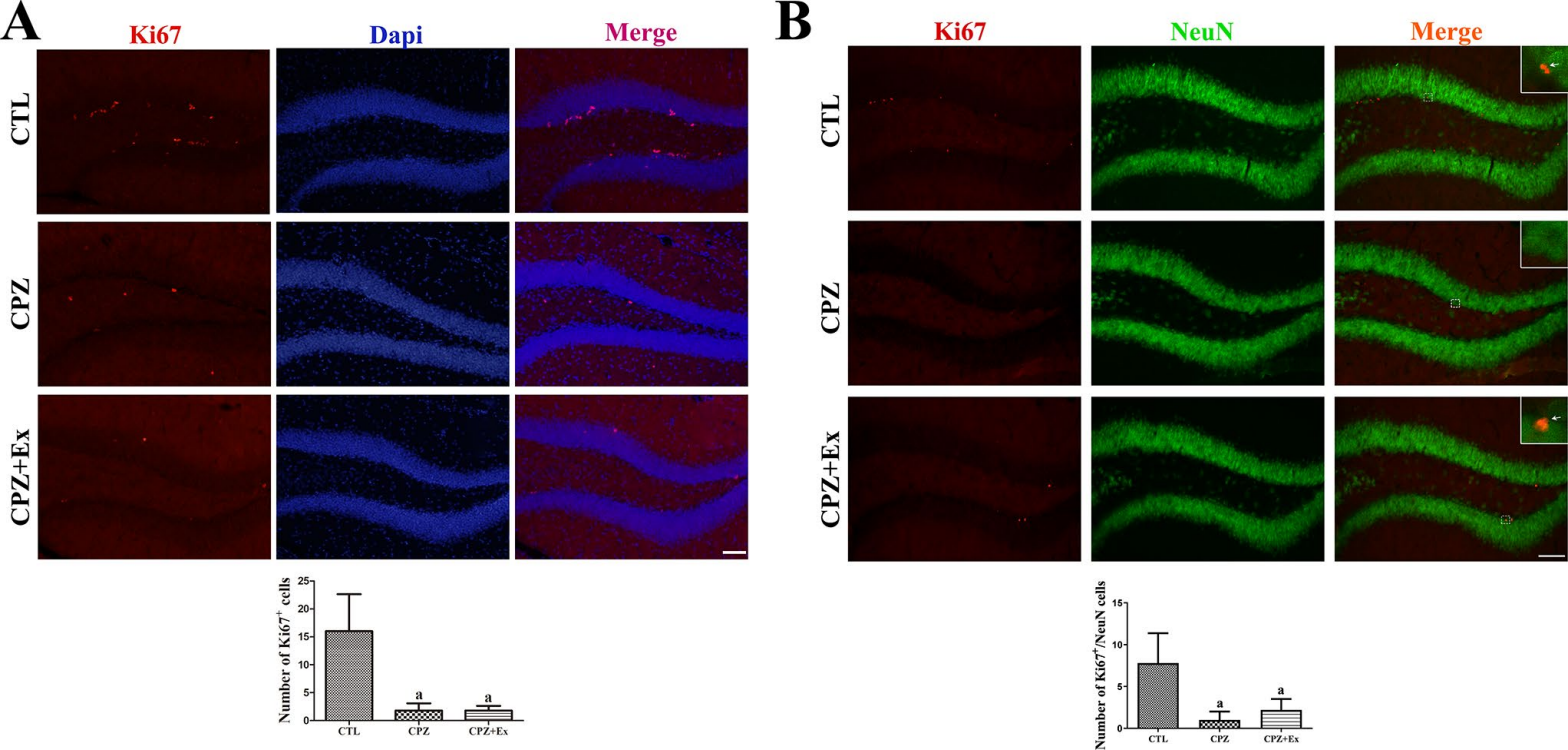
Immunofluorescence analysis of cell proliferation and neuronal markers in the hippocampal dentate gyrus. (**A**) Ki67 immunostaining (red) with DAPI nuclear counterstaining (blue) reveals proliferating cells in the dentate gyrus. Scale bar = 50 μm. (**B**) Double immunofluorescence staining for NeuN (green) and Ki67 (red) in the hippocampal subgranular zone. Scale bar = 75 μm. Insets show higher magnification views. Arrows indicate cells co-expressing Ki67 and NeuN in the merged images. Co-localization of NeuN and Ki67 indicates that a subset of proliferating cells is differentiating into mature neurons. Quantification of both panels (A and B) using Tukey’s multiple comparisons test showed significantly reduced numbers of Ki67-positive cells in the CPZ and CPZ + Ex groups compared to the CTL group (^a^*p* < 0.05 vs. CTL). Error bars represent standard deviation of the mean

To assess hippocampal cell proliferation, Ki67 immunofluorescence staining was performed in combination with 4′,6-diamidino-2-phenylindole (DAPI) (Fig. 6A). In all groups, Ki67-positive cells were predominantly located in the subgranular zone (SGZ) of the dentate gyrus. In the CTL group, the average number of Ki67-positive cells per section was 16.0. However, in both the CPZ and CPZ + Ex groups, the number of Ki67-positive cells was significantly lower, averaging 1.8 per section. This marked decrease suggests that cuprizone administration severely impaired neurogenesis. Furthermore, no significant difference was observed between the CPZ and CPZ + Ex groups, indicating that low-intensity exercise did not significantly counteract cuprizone-induced suppression of cell proliferation.

Double immunofluorescence staining revealed the presence of both Ki67-positive proliferating cells and NeuN-positive mature neurons in the subgranular zone of the hippocampus. As shown in Fig. 6B, merged images indicated co-localization of Ki67 and NeuN, suggesting that some proliferating cells in the SGZ were differentiating into mature neurons within the dentate gyrus. In the Control group, Ki67-positive cells were primarily localized to the SGZ, and NeuN-positive cells were observed in the granular cell layer of the dentate gyrus. The average number of Ki67/NeuN double-positive cells in the CTL group was 7.8 ±, which was significantly higher compared to the CPZ group (1.0 ±, *p* < 0.05) and the CPZ + Ex group (2.2 ±, *p* < 0.05). While the CPZ + Ex group exhibited an increase in Ki67/NeuN double-positive cells compared to the CPZ group, this difference did not reach statistical significance.

### Changes in Immature Neurons in the Hippocampal Dentate Gyrus Following low-intensity Physical Exercise in Cuprizone-Treated Mice

**Fig. 7:**
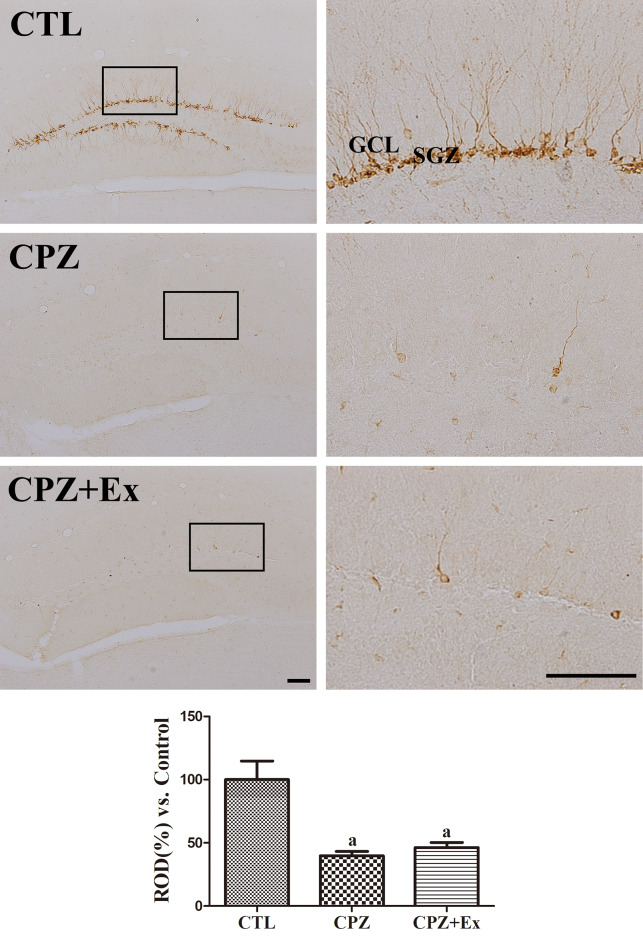
DCX immunohistochemistry in the hippocampal dentate gyrus. High-magnification images show DCX-positive cell bodies in the SGZ with dendrites extending into the GCL. SGZ: subgranular zone; GCL: granular cell layer. Scale bars = 50 μm and 140 μm. Tukey’s multiple comparisons test revealed that the CPZ and CPZ + Ex group showed significant reduction of DCX immunoreactivity when compared to CTL group. However, no significant difference was observed between the CPZ and CPZ + Ex groups. ^a^*p* < 0.05 vs. CTL. The error bars represent the standard deviation of the mean

DCX immunostaining was used to assess immature neurons in the dentate gyrus (Fig. 7). In the CTL group, extensive DCX expression was observed within the subgranular zone (SGZ) and granular cell layer (GCL) of the dentate gyrus. However, both the CPZ and CPZ + Ex groups exhibited significantly reduced DCX expression.

Quantitative analysis revealed that relative optical density (ROD) values for DCX were 39.80% in the CPZ group and 46.33% in the CPZ + Ex group, compared to the CTL group. Despite the slight increase in the CPZ + Ex group, no significant difference in DCX immunoreactivity was observed between the CPZ and CPZ + Ex groups. These findings suggest that while exercise may provide some benefit, it is not sufficient to fully restore neurogenesis in cuprizone-treated mice.

## Discussion

Numerous studies have investigated the effects of physical exercise on neurogenesis in the adult brain [24–26]. Exercise is known to stimulate the formation of new neurons in multiple brain regions [27]. For example, Inoue et al. (2015) demonstrated that low-intensity exercise (15 m/min, below the lactate threshold) more effectively promoted adult hippocampal neurogenesis compared to high-intensity exercise (40 m/min, above the lactate threshold) [28]. Similarly, findings from Aguiar et al. suggest that even low-intensity exercise enhances spatial learning and memory [29]. In this study, we investigated whether low-intensity physical exercise could mitigate cuprizone-induced demyelination and neuroinflammation. By employing a low-intensity forced wheel-running model in mice, we sought to determine whether such exercise could counteract the detrimental effects of cuprizone exposure.

Rodent studies indicate that forced exercise, such as treadmill running or wheel running, induces neuroplastic changes in the hippocampus [30], including increased adult neurogenesis [31], enhanced dendritic arborization [32], and greater synaptic plasticity [33]. On the other hand, voluntary wheel running offers advantages such as alignment with innate animal behavior, reduced stress conditions, and minimal researcher interference, thereby leading to robust physiological adaptations such as enhanced endurance and prevention of sarcopenia [34].

Our study confirmed that cuprizone administration significantly decreased body weight, which aligns with findings from previous research [35, 36]. However, low-intensity physical exercise did not substantially influence this cuprizone-induced weight loss. Although exercise provides various health benefits, our data suggest that the observed body weight reduction was primarily due to the systemic effects of cuprizone rather than the exercise intervention itself.

Immunohistochemical examination of hippocampal tissues revealed patterns consistent with previous reports [20]. Specifically, cuprizone-treated mice showed significantly reduced expression of MBP, Ki67, and DCX in the hippocampus compared to sedentary controls [20]. Moreover, cuprizone administration resulted in morphological activation of microglia, as indicated by the increased Iba1 immunoreactivity characterized by hypertrophied cytoplasm and thickened cellular processes. This activation pattern likely reflects the neuroinflammatory responses associated with cuprizone-induced damage [37].

Consistent with other research indicating the benefits of exercise on remyelination [16], our findings demonstrate that low-intensity forced wheel exercise substantially increased MBP expression in the hippocampal regions, including the alveus, SLM, and corpus callosum. This suggests that low-intensity exercise promotes remyelination following cuprizone-induced damage. These observations align with previous findings from Cheng et al., who reported that forced treadmill exercise augmented MBP expression in the penumbra and mitigated demyelination following focal cerebral injury [38]. Additional studies have also reported increased myelin-related protein expression following treadmill exercise, further supporting the beneficial effects of exercise on myelination [39, 40].Nevertheless, these previous studies utilized high-intensity exercise regimens, whereas our study uniquely demonstrated similar neuroprotective effects using a low-intensity exercise paradigm.

In the current study, we observed significant reductions in microglial activation, suggesting that low-intensity exercise exerts anti-inflammatory effects within the hippocampus. This aligns with previous research by Nakanishi et al., who reported that low-intensity exercise suppressed the pro-inflammatory M1 phenotype of microglia and decreased Alzheimer’s disease pathology, including amyloid-beta deposition and neuronal loss [41]. Our findings are also consistent with other studies demonstrating the beneficial effects of exercise on microglial activation and neuroinflammation [42, 43]. To ensure the reliability of our observations, extensive control measures were implemented, including habituation to handling to minimize stress, maintaining consistent dietary conditions, and providing a stable environment.

Prior research has also highlighted potential mechanisms underlying exercise-induced modulation of microglial morphology, such as reduced inflammatory signaling and enhanced neurogenesis [44]. As TNF-α is a key pro-inflammatory cytokine produced by activated microglia and is implicated in the pathogenesis of various neurodegenerative diseases, including MS [45, 46]. In this study, we investigated TNF-α expression in microglia across hippocampal subregions using double immunofluorescence staining with Iba1 and TNF-α.

Our results demonstrate a significant CPZ-induced increase in TNF-α expression and its colocalization with Iba1⁺ microglia, indicating widespread microglial activation. This effect was most prominent in the CA1 region, consistent with regional vulnerability to inflammation. The associated morphological changes, such as hypertrophic and amoeboid shapes, further support the activated state of microglia in the CPZ group. Although TNF-α levels remained elevated in the CPZ + EX group compared to controls, they were notably reduced relative to the CPZ group, particularly in the CA1 region where a significant decrease in TNF-α/Iba1 colocalization was observed. These findings suggest that voluntary exercise may attenuate microglial inflammation, in part, by modulating TNF-α expression.

Furthermore, colocalization analysis revealed significantly higher TNF-α expression in Iba1-positive cells in both CPZ and CPZ + EX groups compared to controls, highlighting the continued inflammatory potential of microglia even following exercise intervention. This differential regulation suggests that exercise may selectively influence certain aspects of microglial activation while leaving others, such as cytokine production, relatively unaffected.

While activated microglia represent a major source of TNF-α the persistence of TNF-α despite reduced Iba1 expression implies contributions from other CNS cells such as astrocytes or infiltrating immune cells [47]. Temporal dynamics may also account for these findings; previous studies indicate that morphological changes in microglia may precede reductions in cytokine production [48]. Additionally, exercise may induce a shift toward an intermediate microglial state that retains cytokine expression while modifying other functional properties, as observed in Alzheimer’s models [49].

This dissociation between morphology and cytokine levels parallels findings by Xu et al., who reported that specific signaling pathways independently regulate microglial morphology and inflammatory output [50]. The high colocalization of TNF-α with Iba1 in both CPZ and CPZ + EX groups suggests that while microglial density may decline, remaining activated microglia maintain robust TNF-α expression. These findings underscore the complexity of microglial responses and suggest that broad suppression of microglial activation may not suffice to reduce pro-inflammatory cytokine levels. Instead, targeted interventions focusing on cytokine-specific regulatory mechanisms—such as the NLRP3 inflammasome or MAPK pathways—may offer greater therapeutic efficacy [51, 52]. Future studies should investigate whether alternative exercise regimens or combined therapeutic approaches could better modulate inflammatory responses in demyelinating disorders.

Recent evidence underscores the significant role of microglial innate immune memory in influencing neurological disease pathology. As the resident immune cells of the central nervous system (CNS), microglia can acquire a memory-like state in response to peripheral inflammatory stimuli such as lipopolysaccharide exposure. This phenomenon, mediated through mechanisms of trained immunity and immune tolerance, involves epigenetic reprogramming that alters microglial function over the long term [53, 54]. Trained immunity may result in an enhanced pro-inflammatory response upon re-exposure to stimuli, while immune tolerance is associated with diminished reactivity [55]. These persistent changes in microglial activation can significantly affect CNS homeostasis and contribute to the progression of neurological disorders [53, 54]. For instance, trained microglia have been shown to exacerbate neurodegeneration in Alzheimer’s disease by facilitating β-amyloid accumulation and sustaining chronic neuroinflammation [55, 56]. In contrast, microglial immune tolerance may confer neuroprotective effects by attenuating inflammatory responses under certain conditions. In the cuprizone model employed in this study, peripheral inflammatory cues could influence microglial activation states, thereby modulating processes such as hippocampal demyelination and adult neurogenesis [55, 57]. Exploring how microglial innate immune memory interacts with exercise-based interventions may offer novel insights into therapeutic strategies for neurological diseases like MS, where the interplay between peripheral immune activation and CNS pathology is of critical importance [53, 58].

Interestingly, our low-intensity exercise intervention did not significantly improve neurogenesis markers, such as DCX and Ki67, which might be attributed to the severity of cuprizone-induced damage. It is possible that the moderate intensity of our exercise regimen was insufficient to stimulate neurogenesis significantly, given that more vigorous physical activity has been associated with more pronounced neuroplastic adaptations [31]. Indeed, while cuprizone administration significantly impaired hippocampal neurogenesis, low-intensity physical exercise did not effectively reverse this effect.

However, in our study, low-intensity physical exercise did not significantly improve markers of neurogenesis such as DCX and Ki67. This discrepancy could be attributed to the severity of cuprizone-induced hippocampal damage, which might have been too extensive to be significantly reversed by the moderate-intensity exercise employed. Indeed, previous research has shown that higher-intensity exercise regimens are typically more effective at eliciting neuroplastic adaptations [59]. While cuprizone administration significantly impaired hippocampal neurogenesis, low-intensity exercise was insufficient to fully counteract this effect in our model.

The co-expression of Ki67 and NeuN in our study indicates that actively dividing cells are differentiating into neurons, a process crucial for maintaining neural plasticity and cognitive function [60]. This finding is particularly relevant in the context of the cuprizone model, where neurogenesis is often compromised due to demyelination and neuroinflammation [61]. Distinguishing newly formed neurons from other dividing cells, such as glial cells, is essential for accurate interpretation of neurogenesis data [62]. The use of NeuN helps confirm the neuronal identity of Ki67-positive cells, ensuring that the observed neurogenesis reflects neuronal differentiation rather than glial cell proliferation. This specificity is crucial for understanding the true impact of our interventions on neuronal populations and their potential to restore or enhance neurogenesis in conditions where it is impaired.

The observed differences in Ki67+/NeuN + cells following exercise suggest that physical activity can induce neuroplastic adaptations by promoting neurogenesis. This modulation could have significant implications for hippocampal function and behavior, given the well-established link between neurogenesis and learning and memory [60]. Further investigation into the mechanisms underlying these effects could provide insights into how to enhance or restore neurogenesis in conditions where it is impaired, such as neurodegenerative diseases or psychiatric disorders.

Our study highlights several limitations that warrant consideration. First, we exclusively used male mice to control variability associated with the estrous cycle and to maintain consistency with prior research. However, sex differences play an important role in neuroinflammation and demyelination processes [34, 63], emphasizing the need for future studies incorporating both male and female subjects to improve generalizability and explore potential sex-specific therapeutic strategies.

Second, we evaluated only one intensity of exercise, limiting our ability to compare the differential effects of various exercise regimens. The expression of brain-derived neurotrophic factor, known to increase myelin component proteins, is influenced by exercise intensity. Although low-intensity exercise has been shown to promote neurogenesis in the hippocampus, its effect may be less pronounced than that of higher-intensity exercise [64]. Future studies should explore a range of exercise intensities to determine the optimal conditions for neuroprotection and remyelination in demyelinating conditions.

Third, our study specifically focused on the hippocampus, while demyelination affects multiple brain regions. Examining other areas, such as the cerebellum, cortex, or spinal cord, could provide a more comprehensive understanding of how exercise influences neuroprotection across the central nervous system.

Additionally, we assessed only short-term exercise effects. Long-term studies are necessary to elucidate the cumulative or delayed benefits of low-intensity exercise on neurogenesis, remyelination, and neuroinflammation in demyelinating conditions.

Finally, we acknowledge the absence of rescue experiments, which could have provided deeper mechanistic insights into functional recovery. Future studies should investigate whether the neuroprotective effects of exercise persist after cessation or if additional interventions, such as pharmacological treatments or neurotrophic factor administration, could enhance recovery. Exploring the optimal duration and intensity of exercise in combination with other therapeutic strategies will be crucial for translating these findings into clinical applications.

In conclusion, our study demonstrates that low-intensity physical exercise enhances remyelination and modulates neuroinflammatory processes in a cuprizone-induced demyelination model. Specifically, low-intensity exercise significantly increased MBP expression and reduced microglial activation, indicating beneficial neuroprotective effects. Although exercise did not significantly impact neurogenesis markers, these findings highlight its potential therapeutic role for managing demyelinating neurological disorders. Future research should further explore the detailed mechanisms underlying these effects and evaluate different intensities and durations of exercise interventions to maximize clinical relevance.

## Data Availability

No datasets were generated or analysed during the current study.
